# Feasibility of Emergency Department-Initiated HIV Pre-Exposure Prophylaxis

**DOI:** 10.5811/westjem.33611

**Published:** 2024-10-22

**Authors:** Ezra Bisom-Rapp, Kishan Patel, Katrin Jaradeh, Tuna C. Hayirli, Christopher R. Peabody

**Affiliations:** * University of California, San Francisco School of Medicine, San Francisco, California; † University of California San Francisco, Department of Emergency Medicine, San Francisco, California; ‡ Zuckerberg San Francisco General Hospital and Trauma Center, Department of Emergency Medicine, San Francisco, California; § Harvard Medical School, Boston, Massachusetts

## Abstract

**Introduction:**

Pre-exposure prophylaxis (PrEP) for HIV—using antiretroviral medication in non-infected individuals to prevent HIV—has immense potential to slow the spread of the virus. However, uptake has been insufficient, and stark racial disparities exist in both HIV acquisition and PrEP usage, making PrEP access a health equity issue. A promising venue to engage high-risk populations in PrEP care is the emergency department (ED); however, existing ED PrEP initiatives have been costly or have had limited success. We hypothesize that two strategies could overcome these barriers: prescribing PrEP during an ED visit and providing patients with an initial supply of PrEP medication in the ED. Here, we describe the results of a qualitative study exploring multidisciplinary emergency clinicians and HIV clinicians’ needs and views about the feasibility of such an initiative.

**Methods:**

We conducted 22 semi-structured interviews with multidisciplinary clinicians from an urban, safety-net medical center in the ED and the on-site HIV clinic that provides PrEP services. We performed thematic analysis to summarize challenges and potential solutions described by participants.

**Results:**

Participants’ responses fell into three thematic categories: operational challenges; patient-level considerations; and potential impacts. Operational challenges highlighted the difficulty of PrEP initiation in a busy ED and clinician support needs. Patient-level considerations included the complex psychosocial needs of ED patients who could benefit from PrEP. Finally, participants anticipated that an ED-based PrEP initiation program could positively impact both individual patients and public health.

**Conclusion:**

Interviews with emergency department and HIV clinic staff revealed important considerations and potential solutions for ED-initiated PrEP workflows. Clinicians in both specialties were enthusiastic about such an initiative, which could facilitate its success. This study lays the groundwork for the future design of an efficient and innovative workflow to leverage the ED as an essential entry point into HIV prevention services.

Population Health Research Capsule What do we already know about this issue?

*The emergency department is a promising venue for the initiation of HIV pre-exposure prophylaxis (PrEP) for underserved populations.*

What was the research question?

*What challenges and facilitators exist for the creation of an ED-based PrEP initiation and care linkage program?*

What was the major finding of the study?

*Busy EDs with limited clinician support and patients’ complex psychosocial needs are factors to consider in the creation of an ED-based PrEP initiative.*

How does this improve population health?

*Prescribing PrEP during a patient’s initial ED visit before receiving all lab results could increase the likelihood of PrEP initiation and continuation in marginalized communities.*

## INTRODUCTION

Despite substantial progress in understanding and managing human immunodeficiency virus (HIV), the virus remains a pressing concern in the United States due to its persistent prevalence, associated multisystemic health impacts, and costs to the healthcare system.^1^ ^–^ ^4^ This underscores the need for innovative approaches to prevention. HIV pre-exposure prophylaxis (PrEP)—the use of antiretroviral medication in non-infected individuals to prevent HIV acquisition—has emerged as a groundbreaking strategy that drastically reduces the risk of contracting HIV and has received a Grade A recommendation from the US Preventative Services Task Force.^5^ ^,^ ^6^

While PrEP is a safe and effective HIV prevention tool, challenges persist that have limited its real-world impact on the HIV epidemic. Uptake has been poor: just 36% of people at risk for HIV with a PrEP indication were prescribed the medication in 2022.^7^ Wide racial and socioeconomic disparities exist in both HIV acquisition and PrEP uptake in the US,^8^ ^–^ ^10^ leaving vulnerable populations disproportionately exposed to the risk of HIV infection. Addressing these disparities necessitates innovative approaches that extend beyond traditional sexual health clinic settings. Emergency departments (ED) are often the only healthcare access point for underserved populations and have been identified as a promising venue to engage high-risk populations in HIV prevention services.^11^ Research in an urban, county-run ED estimates that at least 1 in 20 ED patients are PrEP-eligible, and of the PrEP-eligible participants who had previously heard of PrEP, 75% were willing to start at that visit.^12^ Other studies have similarly demonstrated PrEP’s acceptability among ED patients.^13^ ^,^ ^14^

While many ED patients could benefit from PrEP, connecting them to longitudinal care at PrEP clinics remains challenging. A study in a high-volume urban ED showed that while 68.6% of patients who completed an HIV risk assessment were interested in PrEP, 11% of those interested were able to start PrEP medication after speaking with a PrEP educator, and 3% of interested patients who were provided with information about drop-in PrEP clinic hours received a prescription.^14^ Other EDs have similarly implemented ED-based PrEP educators or navigators with varying degrees of success.^15^ ^,^ ^16^ However, this strategy may be cost-prohibitive for many safety-net EDs, as they may not be able to fund or staff PrEP-specific positions to assist with linkages. More work is needed to determine how to address low PrEP initiation rates, while leveraging existing resources and interdisciplinary personnel to provide equitable, high-value care.

We have identified two strategies studied in other contexts that could be adapted to address barriers faced by previously reported ED-based PrEP programs. First, research in drop-in sexual health clinics shows that prescribing PrEP during a patient’s initial visit (“same-day PrEP initiation”), before receiving all lab results, increases the likelihood of PrEP initiation and continuation without compromising patient safety, compared to the standard model of requiring multiple visits for testing and counseling before prescribing PrEP medication.^17^ Second, providing a 14-day supply of antiretroviral medication “starter packs” to patients who test positive for HIV in the ED has been shown to increase the likelihood of engagement in follow-up HIV care, compared to patients who did not receive medication starter packs.^18^ We hypothesize that adapting both of these strategies to create a workflow that employs “same-day PrEP initiation” within the initial ED visit and PrEP “starter packs” could overcome challenges and financial barriers faced by previously described programs solely focused on counseling and referral to services. However, the feasibility and acceptability of such a workflow among ED and HIV clinic staff is unknown.

In this paper, we present a qualitative study investigating the feasibility of, and identifying barriers and facilitators to, the implementation of a same-day PrEP initiation workflow in the ED via thematic analysis of interviews conducted with multidisciplinary clinicians from an urban ED and a safety-net HIV clinic that provides comprehensive PrEP services. This exploratory study lays the groundwork for creation and evaluation of such a workflow.

## METHODS

### Study Design and Participant Selection

We conducted 22 half-hour interviews at a large, urban, safety-net medical center in a major California city with 15 multidisciplinary clinicians in the ED and seven in the on-site HIV clinic between May 25–July 12, 2023 and July 15–July 24, 2024. We chose this site because it is a county-run, safety-net institution that has both an ED and HIV clinic on the same campus and serves mainly publicly insured and uninsured patients. Moreover, many of this center’s patients experience psychosocially complex circumstances such as houselessness or substance use disorders and are at high risk for HIV from sharing injection drug equipment, engaging in sex work, or having condomless anal sex with multiple partners. We identified participants by purposive sampling, collaboratively drafting a list of potential subjects to represent a diverse array of professional experiences, years of practice, and degree of past involvement in harm reduction initiatives.

We intentionally recruited subjects who serve in a variety of clinical roles to identify opportunities for interprofessional collaboration and staffing efficiency. Additionally, participants were identified by snowball sampling by asking participants to refer additional staff members who could add a unique perspective to the dataset. Recruitment continued iteratively in this way until thematic saturation was reached, meaning that interviewers agreed that interviews were yielding similar data. Participants received $25 gift cards for their participation. Our institution’s institutional review board determined this study to be exempt.

### Interventions

Interviews were semi-structured, using an interview guide created collaboratively by authors EBR and KJ and iteratively modified by all authors (Supplemental Table 1). The interview guide invited participants to discuss their role in the ED, previous experience with ED-based HIV prevention interventions, perceived need for HIV prevention interventions among their patient population, and anticipated barriers and solutions for successful PrEP initiation and linkage to care. All participants provided verbal consent to participate in the study (Supplemental Table 2). Besides role, no identifying information was collected. All interviewers (EBR, KP, and KJ) have experience creating curriculum or workflows for medical professionals and students on topics related to the needs of marginalized populations. We considered how interviewer positionality could potentially impact participants’ responses; no issues were found, and none of the interviewers had supervisorial responsibilities over the participants they interviewed. EBR conducted 14 interviews; KP conducted six interviews, and KJ conducted two interviews. Interviews were recorded and transcribed using Zoom teleconferencing software v5.13.11 (Zoom Video Communications, Inc, San Jose, CA), and transcripts were reviewed for accuracy by EBR. Recordings and transcripts were stored securely within our institution’s approved secure, enterprise, cloud-based file collaboration software. Sampling and interviews were conducted until no new information was generated, indicating that thematic saturation was reached.

### Analysis

We performed inductive thematic analysis of interview transcripts after completing interviews following the grounded theory approach. Allowing participants’ responses to guide theme development enabled us to prioritize problem areas not already explored in the literature.^19^ Analysis was conducted from July 15–August 15, 2023 and July 25–August 2, 2024. At the initial stage, authors EBR, KP, and KJ inductively developed a preliminary codebook by reflecting on interviews and analyzing one full transcript together. This codebook was iteratively revised during coding in discussion with the research team. EBR coded 12 interviews and KP coded 10 interviews using ATLAS.ti web software v5.11.0-2023-08-02) (ATLAS.ti GmbH, Berlin, Germany). After independently reviewing each other’s codes, EBR and KP reconciled any discrepancies by discussing them within the context of the full interview transcript, and KJ arbitrated disagreements. There was a high degree of concordance between coders, and complete agreement regarding codes and themes was reached. Throughout this process, higher order themes were developed through iterative discussions among the research team. Frequent meetings at each analytic step enhanced consistency of coding and analysis. Funders did not participate in any portion of data collection or analysis.

In this paper, we use “HIV clinic” to describe the study site at which interviews took place, and “PrEP clinic” to indicate any clinic that could form partnerships with EDs to facilitate follow-up PrEP care.

## RESULTS

Our sample included eight attending physicians (five in the ED and three in the HIV clinic); four resident physicians and fellows (three in the ED and one in the HIV clinic); one nurse practitioner in the ED; five registered nurses (three in the ED and two in the HIV clinic); two social workers in the ED; and two pharmacy staff (one in the ED, and one in the HIV clinic) (Table 1).

**Table 1.** Participant demographics among 22 healthcare professionals from the emergency department (ED) and HIV clinic at Zuckerberg San Francisco General Hospital, an urban safety-net medical center.

	Emergency department	HIV clinic	Total
Role			
Attending physician	5	3	8
Resident physician/fellow	3	1	4
Nurse practitioner	1	0	1
Registered nurse	3	2	5
Social Worker	2	0	2
Pharmacist/pharmacy technician	1	1	2
Total	15	7	22
Years of practice			
1–5 years	3	2	5
6–10 years	4	3	7
11+ years	8	2	10
Experience with PrEP in the ED			
None	9	4	13
Limited^*^	5	1	6
Extensive^**^	1	2	3

* Limited PrEP experience is defined as “not within the clinician’s usual scope of practice, but they have been involved in connecting ED patients to PrEP at least once.”

** Extensive PrEP experience is defined as having been “involved in ED-prescribed PrEP multiple times and is comfortable in this practice.”

*ED*, emergency department; *PrEP*, pre-exposure prophylaxis.

Participants self-identified their experience with delivering PrEP as “none,” “limited,” or “extensive.” “Limited” PrEP experience is defined as not within the healthcare professional’s usual scope of practice, but they have been involved in connecting ED patients to PrEP at least once. “Extensive” PrEP experience indicates that they have been involved in ED-prescribed PrEP multiple times and are comfortable in this practice. Thirteen participants had no experience delivering PrEP (nine in the ED and four in the HIV clinic); six had limited experience (five in the ED and one in the HIV clinic); and three had extensive experience (one in the ED and two in the HIV clinic). Themes discussed by participants fell into three distinct categories: operational challenges; patient-level considerations; and potential impacts of an ED-initiated PrEP workflow.

## Operational Challenges

Operational challenges to implementing an ED-based PrEP initiation workflow were reported consistently by participants from both the ED and the HIV clinic (Table 2). The main concerns participants identified were time, capacity, and resource constraints in the ED. Emergency clinicians were wary of initiating conversations about preventative health unrelated to the patient’s chief concern, as doing so could extend ED length of stay and impede ED operations. One emergency physician expressed trepidation about clinicians’ ability to have “meaningful conversations [about sexual health and HIV risk] while trying to care for [many] people in the waiting room at the same time”. Needing to conduct lengthy searches for clinical guidelines from multiple sources was also a recurring factor.

**Table 2.** Representative quotes from participant interviews illustrating operational challenges to implementing an emergency department-initiated pre-exposure prophylaxis workflow.

Sub-themes within operational challenges	Associated quotations
Time and capacity constraints for PrEP care within the ED	“This proposed project is coming at a… national crisis of ED crowding… so there’s really little excess capacity anywhere in, in and outside the ED for additional tasks, without more resources.” (EDMD-01) “We’re usually short staffed, and there’s… competing priorities usually during the shift, and something like this will probably fall to the wayside in terms of priority list, but not to say it’s not important.” (EDRN-3)
Challenges associated with equitably identifying high-need PrEP candidates	“I need [clinicians] to offer this to people, but not just those who[m] you assume are at risk, because… we have a lot of patients who are sex workers, and they don’t necessarily tell us they’re sex workers.” (EDNP-01)
Need for staff education	“I think I would just need a bit more information myself… I have no real expertise in this area. And so, I would definitely want to have a better understanding of… what I was looking for… Then I would feel pretty comfortable having a conversation as long as… I was educated enough.” (EDSW-02)

*ED*, emergency department; *EDMD*, emergency physician; *EDRN*, registered nurse in the ED; *EDSW*, ED social worker; *PrEP*, pre-exposure prophylaxis.

To circumvent these time and capacity constraints, nearly all participants discussed electronic health record (EHR) tools or electronically accessible workflows as factors that could facilitate rapid PrEP prescription and decrease the “knowledge base needed [to correctly prescribe PrEP]” (HIV Clinic physician). The EHR tools included premade order sets to facilitate ordering of all required labs, prescription formulations, and referrals; a checklist of topics to review with patients integrated into medical note templates; and pre-written notes and discharge instructions. Integrating these tools into one page in the EHR or electronically accessible workflow system could allay concerns over the cognitive burden of implementing this workflow that some emergency clinicians considered to be outside their routine scope of practice.

Interdisciplinary collaborations were commonly discussed. The emergency physicians highlighted the importance of interdisciplinary collaborations between physicians, nurses, pharmacists, social workers, and patient navigators, “so that the burden isn’t solely on [one clinician] to explain everything to the patient” (emergency medicine resident). Many felt that this could ease the time and capacity constraints felt by physicians in their daily practice. This sentiment was similarly echoed by the nurses, pharmacists, and social workers interviewed who felt their participation could strengthen the program. One nurse noted that, although discharge planning is coordinated by prescribers, these plans sometimes result “from the advocacy of bedside nurses” (ED nurse); formalizing and encouraging interdisciplinary collaboration could increase patient identification and linkage. However, another nurse noted that putting too much burden on already busy nursing staff would limit the success of the program.

Participants anticipated challenges in identifying patients who would be ideal PrEP candidates. Some worried about the potential “bias of who [clinicians] think is at risk” (ED nurse practitioner), reinforcing stereotypes, or incomplete identification of need based on patient reluctance to disclose risky behaviors. However, many had mixed feelings about the solutions they identified. While many participants suggested automated EHR pop-up reminders triggered by certain chief concerns or charted risk factors, they anticipated that these reminders may be ignored by clinicians inundated by a burgeoning number of similar alerts. Some suggested universal screening for HIV risk factors to avoid biased PrEP offering but anticipated feasibility and privacy issues. Finally, many suggested posters encouraging patients to self-identify as PrEP candidates, but others felt this strategy would miss high-need patients with limited health literacy or reluctance to disclose risk factors.

Participants emphasized the importance of staff education, with many reporting that their comfort with the program would be contingent on adequate staff training. There was no consensus on ideal length and modality of educational session, and some emphasized the need for a variety of training formats—live lectures, team huddles, emails, posters—to meet the needs of staff with a variety of learning styles and schedules. With regard to training content, emergency clinicians desired more information about the potential harm that PrEP therapy could cause patients, particularly related to liver and kidney health. However, HIV clinic staff consistently emphasized that ED staff training should highlight that PrEP carries a low risk for severe injury if clinicians adhere to prescriber guidelines.

### Patient-Level Considerations

In addition to operational concerns, participants frequently reported that an ED-based PrEP initiation program must address factors that influence patients’ ability to enter and maintain engagement with longitudinal PrEP care (Table 3). The need for mechanisms for reliable connection to follow-up care, including plans for patients who present after business hours, was discussed ubiquitously. Multiple HIV clinic participants emphasized collecting alternate forms of contact information that might not usually be asked about during triage, including email addresses, friends’ or case workers’ contact information, and campsite locations for unhoused patients. Many from both the ED and HIV clinic emphasized the importance of having a clearly defined follow-up structure or specific person to conduct outreach to patients with abnormal lab results that resulted after discharge from the ED.

**Table 3.** Representative quotes from participant interviews illustrating patient-level considerations when implementing an emergency department-initiated pre-exposure prophylaxis workflow.

Sub-themes within patient-level considerations	Associated quotations
Ensuring adequate follow-up	“In terms of obtaining good contact information from people—and I don’t just mean phone numbers by that. So, it could be campsites, places they frequent, friends’ contact numbers if somebody doesn’t have a reliable means of communication, because we certainly don’t want to create barriers for the people who need PrEP the most.” (IDMD-03)
Anticipated barriers to accessing and adhering to medications	“I do worry that many patients who are at risk for HIV may not have the faculties to be able to take a daily medication.” (EDMD-05)

*EDMD*, emergency physician; *IDMD*, infectious diseases physician; *PrEP*, pre-exposure prophylaxis.

Patient navigators were discussed as an essential resource to facilitate connection to care. Multiple participants from the HIV clinic mentioned the value of having a navigator based at the HIV clinic meet the patient in the ED, as opposed to relying on ED-based support staff for connection to care. They felt that this could build therapeutic rapport, decrease patient anxiety associated with seeking services in a new healthcare setting as “the ice is already broken” (HIV Clinic physician), and increase likelihood of retention in care.

Due to the psychosocial complexity of the target patient population, participants proposed three components of a PrEP initiation workflow that are essential for success. First, many participants discussed the need to provide patients an initial supply of PrEP in the ED, as opposed to sending the prescription to a pharmacy for the patient to pick up after discharge. Participants reported having experienced greater success in discharge medication initiation when this strategy was employed in similar initiatives. Second, participants emphasized the importance of EDs partnering with PrEP clinics that could meet additional psychosocial needs “that are really at the forefront of [the patient’s] life” (HIV Clinic nurse). For example, many participants from both the ED and HIV clinic anticipated that a PrEP clinic that provides substance use disorder support and wrap-around services could better address barriers to adherence in high-risk patients than a clinic that only offers basic sexual health care. Third, participants discussed the importance of language-inclusive patient education materials and staff for patients with limited English proficiency, as well as patient education materials that use simple language.

## Potential Impacts

Participants near universally anticipated a high need for an ED-based PrEP initiation workflow, and the majority were noticeably excited for the potential rollout of such an initiative (Table 4). Participants stated that this initiative could have a significant impact on patients who are at high risk for HIV infection and have high levels of psychosocial complexity, especially unhoused patients and those with substance use disorders. Furthermore, participants anticipated that this program could have positive implications on population-level health disparities in HIV acquisition by reaching high-risk groups that current HIV prevention initiatives have not been able to engage.

**Table 4.** Representative quotes illustrating participant-reported potential impacts of an emergency department-initiated pre-exposure prophylaxis workflow.

Sub-themes within Potential Impacts	Associated quotations
Individual and public health benefits of engaging at-risk populations in HIV prevention services	“A lot of people come to our emergency department, I say sometimes as a last resort, but also as a first resort, because they don’t know where else to go… and I think that it will touch a lot more people than I think we think it will at this point.” (EDRN-01)
Impacts on staff	“I think that [clinicians] are going to love being able to provide people with a wellness act instead of meeting people in their moment of crisis. Because that’s what we do all the time. So how great is it that we can actually help people that want to help themselves to stay healthy.” (EDRN-01)
Differing views on the ED’s evolving role in the healthcare system	“Some people literally don’t see this as the role of the ER, and it’s going to take some, like, arm twisting.” (EDNP-01) “And especially here the context of… a community hospital, … a lot of people who choose to work here know that [community protection is] part of our emergency department job.” (EDRN-02) “I think most people in emergency medicine recognize that our role as emergency physicians is constantly expanding and contracting in relationship to what is happening in the world.” (EDMD-04)

*ED*, emergency department; *EDMD*, emergency physician; *EDNP*, nurse practitioner in the ED; *EDRN*, registered nurse in the ED.

Participants had differing views on the effect that this program could have on ED staff and operations. Both ED and PrEP clinic participants worried that emergency clinicians would resist the additional tasks required for this program and find it incompatible with an acute care setting. One emergency attending physician, speaking to her previous experience leading an ED-based harm reduction initiative, noted that considerable educational interventions were needed to “change the [ED] culture and make [prescribing harm reduction medication] something that was within the purview of the emergency team.” However, many ED staff anticipated that their colleagues would find fulfillment in the opportunity to engage patients in preventative medicine as a respite from managing frequent acute crises. Additionally, some ED participants viewed this proposed workflow as part of a larger perceived culture shift in EDs to consider upstream factors that cause patients to seek out acute care, even fulfilling a moral obligation: “We owe it to our community to be able to provide [PrEP in the ED]” (emergency medicine resident).

## DISCUSSION

This study demonstrated that staff at both a busy, urban medical center ED and safety-net HIV clinic see the need for HIV prevention services among ED patients and are amenable to the creation of a workflow to engage at-risk patients in care while in the ED, assuming that described challenges are addressed. We integrated themes that arose into a cohesive framework that could be used to guide workflow development (Figure).

**Figure.**
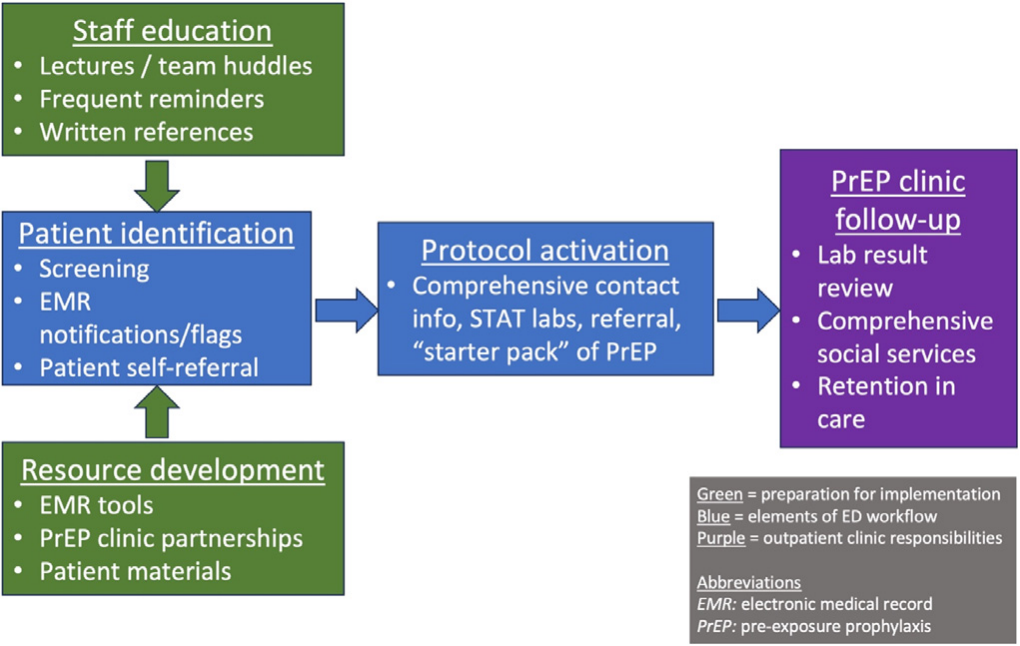
Themes integrated into a cohesive framework to guide the creation and implementation of an emergency department-based PrEP initiation workflow. *ED*, emergency department; *EHR*, electronic health record; *PrEP*, pre-exposure prophylaxis.

Our data provides valuable insight into potential interventions to prepare and prime ED staff to consider PrEP as an option for patients. For example, discordant views on PrEP safety and side effects between ED and HIV clinic staff demonstrate the need for educational interventions targeted at this subject. Furthermore, the perspectives of ED staff excited about the potential workflow—such as finding fulfillment in connecting patients with PrEP or evoking a moral obligation among staff—could be incorporated into interventions designed to shift ED culture to be more amenable to prescribing PrEP.

In our interviews, EHR tools surfaced as a promising intervention to address clinician concerns. Clinicians felt that order sets and templates could decrease the amount of time required for clinicians to find and follow up-to-date guidelines. Indeed, EHR templates have been shown to improve clinical guideline adherence for other health issues.^20^ ^,^ ^21^ Electronic platforms that allow clinicians to access standardized workflows quickly, which emergency clinicians commonly requested, could further supplement these tools to increase clinician confidence with the program.^22^

Additionally, clinicians’ concerns over equitable identification of PrEP candidates, as well as trepidation about EHR-based pop-up HIV risk alerts, are reflected in research. A study conducted in a large, managed care health system showed that an EHR-based HIV risk predictive model based solely on PrEP guidelines from the Centers for Disease Control and Prevention (CDC) was less sensitive in identifying HIV risk in Black patients compared to White patients, whereas a multivariable algorithm generated through machine learning was equally sensitive among races.^23^ This demonstrates the potential for EHR tools to ameliorate racial disparities in PrEP offer rate, as well as the need for thoughtful design of these tools to avoid exacerbating health disparities.

Whereas previous studies about PrEP in the ED focused only on referral to services,^11^ ^,^ ^14^ ED and HIV clinic staff both anticipated that immediate PrEP initiation with a starter pack provided in the ED would increase chances of follow-up. This prediction is supported by existing literature; immediate PrEP initiation at the patient’s first visit has been shown to facilitate increased PrEP uptake and persistence in drop-in sexual health clinic settings and is described as a tool to address PrEP access barriers in the 2021 CDC PrEP guidelines.^5^ ^,^ ^17^ This strategy merits further investigation for adaptation to an acute care setting.

Furthermore, our data supports a partnership model between the ED and a single outpatient clinic. This could streamline the referral process and decrease costs by removing the need for an ED-based PrEP navigators or educators to guide patients through complex healthcare systems. Direct “warm handoffs” to PrEP clinic-based navigators could also facilitate the early development of therapeutic rapport with patients and promote retention in care, addressing the low-yield of programs based on providing resource sheets with PrEP clinic information.

As noted by participants, the services of the partnering PrEP clinic should match the needs of the target population served by the ED, including treatment for substance use disorder in localities disproportionately affected by the opioid epidemic. Additionally, EDs must equip their partnering PrEP clinics with the information needed to facilitate successful connection to the specific services provided by that PrEP clinic. For example, PrEP clinics that perform outreach to the unhoused may need information such as the location of patients’ encampments, whereas clinics that do not offer these outreach services may not find this information useful. The local context of both services needed and services available should be taken into consideration when designing an ED-initiated PrEP program.

## LIMITATIONS

To our knowledge, this exploratory study is the only one of its kind to evaluate the needs and viewpoints of multidisciplinary clinicians regarding an ED-initiated PrEP workflow. However, our study does not capture the patient perspective on a program in which a patient would initiate PrEP in the ED, an important topic for future research. Additionally, this study was conducted at a large medical center in a city with a robust social safety net and may not be generalizable to smaller community hospitals.

## CONCLUSION

Our study describes anticipated challenges and facilitators of initiating of a pre-exposure prophylaxis workflow in the ED from the perspective of multidisciplinary emergency and HIV-clinic clinicians. The perspectives of the multidisciplinary participants interviewed are essential for developing a comprehensive, successful workflow. Recommendations described here provide a framework for the creation of a novel PrEP initiation program. Collaborations between the ED and preventative medicine programs may have profound implications for health equity as acute care facilities expand their role in the community to facilitate access to preventative care to those who have no other source for these services.

## Supplementary Information




