# Supplementary material for: Feasibility of Emergency Department-Initiated HIV Pre-Exposure Prophylaxis
Source: West J Emerg Med. 2024 Oct 22;25(6):985–92. doi: 10.5811/westjem.33611 (PMC11610728; doi:10.5811/westjem.33611)
Supplement: Supplementary file 1 [file wjem-25-985-s001.docx]

**Supplemental Table 1**: Interview guides used for this study, separated by job title and practice location of participant.

| Position title and practice location | Interview guide for the corresponding job title |
| --- | --- |
| Emergency Department Attending and Resident Physicians, and Nurse Practitioners | Pre-Exposure Prophylaxis for HIV (PrEP) is a daily pill (or injection administered every two months) that’s 99% effective at preventing HIV. Emergency Departments have been described as promising locations to identify PrEP candidates due to the high volume of patients, many of whom are not engaged in Primary Care and experience risk factors such as injection drug use, transactional sex, and houselessness. We are conducting a needs assessment to evaluate the feasibility of implementing a PrEP initiation and connection-to-care program at the [HOSPITAL] Emergency Department (ED). ED providers would identify patients at high risk for HIV, order labs and an initial oral PrEP prescription, and provide a direct referral to [HIV CLINIC] PrEP navigators. These PrEP navigators would then link the patients to ongoing care in their neighborhood of choice. The purpose of this interview is to get your perspective on the potential creation and implementation of the program. With your consent, our conversation today will be transcribed and recorded. Thank you for your participation in this study.  Background:   1. Tell me about your role in the ED. 2. How long have you been in practice?    1. (Code by 5 yrs) 3. How long have you been working at [HOSPITAL]?   Experiences with prescribing PrEP: Now, I’d like to hear a little more about your past experiences with PrEP. As we discussed, PrEP is a safe and effective medication that prevents HIV.   1. Do you have any experience prescribing PrEP? 2. If yes, how did it go? 3. Do you know a colleague who has?   Current thoughts about/comfort with PrEP: Let’s talk about your current thoughts about PrEP.   1. Do you feel PrEP could be a helpful intervention for some of your patients? Why? 2. Currently, how comfortable are you identifying patients who might be eligible for PrEP? How comfortable prescribing PrEP? Linking patients to care afterwards? 3. Tell me a little more about that.   Creation and implementation of workflow: Imagine that we develop a standard workflow for prescribing PrEP in the ED.   1. What would make a successful PrEP initiation program? *Move on to sub-points only if participant does not have sufficient suggestions.*    1. Provider familiarity:       1. Regular training? What would be the ideal format and length?       2. Scripts for providers?    2. Prescribing logistics:       1. Dot phrases? Order sets in Epic? E-drive link?       2. Pop-up prompts in Epic to consider PrEP when ordering STI tests?    3. Patient education and follow-up       1. Patient hand-outs or AVS dot phrases?       2. Direct handoffs to [HIV CLINIC] PrEP navigators vs Epic referrals? 2. Research shows that prescribing PrEP during the patient’s initial visit (i.e. before all lab results come back) is safe and greatly increases the likelihood that a patient will complete a follow-up appointment in drop-in sexual health clinic settings. Would you be comfortable discharging a patient from the ED with an initial prescription? Why? 3. What challenges do you anticipate with implementing a new workflow for prescribing PrEP in the ED?    1. How could they be overcome?   Anticipated future comfort levels and close-out:   1. If this workflow was implemented as we discussed, how comfortable do you think you’d be prescribing PrEP to patients with PrEP indications? 2. How realistic do you think an ED-based PrEP initiation and linkage program would be?    1. *If participant is unsure:* Do ED clinicians have capacity, assuming patient volume is low (< 3 patients identified per month)? 3. *For resident physicians or fellows only*: Would you feel comfortable discussing PrEP with your attending if you identified a patient with PrEP indications? 4. Is there anything else you would like me to know? 5. Do you have any colleagues who you think I should talk to about this project? |
| Emergency Department Nurses, Social Workers, Navigators, and Other Staff | Pre-Exposure Prophylaxis for HIV (PrEP) is a daily pill (or injection administered every two months) that’s 99% effective at preventing HIV among at-risk individuals. Emergency Departments have been described as promising locations to identify PrEP candidates due to the high volume of patients, many of whom are not engaged in Primary Care and experience risk factors such as injection drug use, transactional sex, and houselessness. We are conducting a needs assessment to evaluate the feasibility of implementing a PrEP initiation and connection-to-care program at the [HOSPITAL] ED. ED providers would identify patients at high risk for HIV, order labs and an initial prescription, and provide a direct referral to [HIV CLINIC’S] PrEP navigators. These PrEP navigators would then link the patients to ongoing care in their neighborhood of choice. The purpose of this interview is to get your perspective on the potential creation and implementation of the program. With your consent, our conversation today will be transcribed and recorded. Thank you for your participation in this study.  Background:   1. Tell me about your role in the ED.    1. How long have you been in practice?    2. How long have you been working at [HOSPITAL]?   Experiences and with PrEP: Now, I’d like to hear a little more about your past experiences with HIV prevention and PrEP.   1. Do you screen for social determinants of health? Talk about sexual health? 2. Do you currently help patients access sexual health or HIV prevention resources in your capacity as a provider in the ED? Which resources? How does this usually work? 3. If no, have you ever? 4. Have you ever helped patients access PrEP in any other setting? If yes, how did it go? 5. Do you know a colleague who has?   Comfort level with PrEP: Let’s talk about your current thoughts about PrEP.   1. Currently, how comfortable are you identifying patients who might be eligible for PrEP? Linking patients to care? Tell me a little more about that. 2. Do you feel PrEP could be a useful tool for some of your patients? Which patients? How useful do you think initiating PrEP in the ED would be for these patients? Why?   Creation and implementation of workflow and inter-professional collaboration: Imagine that we develop a standard workflow for prescribing PrEP in the ED.   1. What aspects of a PrEP initiation program would make the program more successful? *(Prompt with suggestions below if participant does not have sufficient material to discuss.)*    1. Dot phrases in Epic specific for your job title? Automatic Best Practice Advisories for patients with indications?    2. Regular training? What would be the ideal format and length? Provider handouts or scripts?    3. Written patient hand-outs? 2. What challenges do you anticipate with implementing this workflow? How might they be overcome? 3. If you identified a patient who had a PrEP indication, would you feel comfortable raising this concern to an ED physician so that they could prescribe the patient PrEP?   Anticipated future comfort levels and close-out:   1. If this workflow was implemented as we discussed, how comfortable do you think you’d be connecting patients to PrEP care? 2. Do you think [interviewee’s job title] has capacity to help patients initiate PrEP, assuming that patient volumes are low (< 3 patients identified per month)? 3. Is there anything else you would like me to know? 4. Do you have any colleagues who you think I should talk to about this project? |
| HIV Clinic Attending and Resident Physicians, and Nurse Practitioners | Emergency Departments have been described as promising locations to identify HIV PrEP candidates due to the high volume of patients, many of whom are not engaged in Primary Care and experience risk factors such as injection drug use, transactional sex, and houselessness. We are conducting a needs assessment to evaluate the feasibility of implementing a PrEP initiation and connection-to-care program at the [HOSPITAL] Emergency Department. ED providers would identify patients at high risk for HIV, order labs and an initial prescription, and provide a direct referral to [HIV CLINIC] PrEP navigators. These PrEP navigators would then link patients to ongoing care at their preferred clinic. The purpose of this interview is to get your perspective on the potential creation and implementation of this workflow. With your consent, our conversation today will be transcribed and recorded. Thank you for your participation in this study.  Background:   1. Briefly tell me about your role at [HIV CLINIC]. 2. How long have you been in practice? 3. How long have you been working at [HIV CLINIC]?   Thoughts about ED-delivered PrEP: Now, let’s talk about PrEP in the ED.   1. Have you or a colleague ever received a referral for PrEP from the ED?    1. If yes, how did it go? 2. Do you feel PrEP could be a helpful intervention for ED patients at risk for HIV? Why?   Creation and implementation of workflow: Imagine that we develop a standard workflow for prescribing PrEP in the ED.   1. Describe what an ideal ED-initiated PrEP program would look like from your perspective.    1. What could help with prescribing logistics?       1. Dot phrases? Order sets in Epic? E-drive link?       2. Pop-up prompts in Epic to consider PrEP when ordering STI tests?    2. What might increase provider familiarity with PrEP?       1. Regular training? What would be the ideal format and length?       2. Scripts for providers?    3. What could facilitate patient education and follow-up?       1. Patient hand-outs or AVS dot phrases?       2. Direct handoffs to [HIV CLINIC] PrEP navigators versus Epic referrals?    4. What challenges do you anticipate with implementing a new workflow for prescribing PrEP in the ED? How could they be overcome?    5. What concerns might you have about the implementation of this program? 2. What would you like ED providers to know before they begin prescribing PrEP? 3. Research shows that prescribing PrEP during the patient’s initial visit (i.e. before all lab results come back) is safe and greatly increases the likelihood that a patient will complete a follow-up appointment in drop-in sexual health clinic settings. Would you be comfortable with ED providers prescribing PrEP? Why?   Anticipated future comfort levels and close-out:   1. If this workflow was implemented as we discussed, how comfortable do you think you would be with this program? Why? 2. Is there anything else you would like me to know? 3. Do you have any colleagues who you think I should talk to about this project? |
| HIV Clinic Nurses, Social Workers, Navigators, and Other Staff | Emergency Departments have been described as promising locations to identify HIV PrEP candidates due to the high volume of patients, many of whom are not engaged in Primary Care and experience risk factors such as injection drug use, transactional sex, and houselessness. We are conducting a needs assessment to evaluate the feasibility of implementing a PrEP initiation and connection-to-care program at the [HOSPITAL] ED. ED providers would identify patients at high risk for HIV, order labs and an initial prescription, and provide a direct referral to ongoing services. The purpose of this interview is to get your perspective on the potential creation and implementation of the program. With your consent, our conversation today will be transcribed and recorded. Thank you for your participation in this study.  Background:   1. Tell me about your role at [HIV CLINIC]. 2. How long have you been in practice? 3. How long have you been working at [HIV CLINIC]?   [HIV CLINIC] PrEP workflow and experience with ED-delivered PrEP: Now, let’s talk about PrEP.   1. Have you or a colleague ever received a referral from the ED for PrEP or HIV care? If yes, how did it go? 2. How helpful do you think ED-initiated PrEP would be for at-risk patients who present to the ED?   Creation and implementation of workflow and inter-professional collaboration: Imagine that we develop a standard workflow for prescribing PrEP in the ED.   1. What aspects of a PrEP initiation program would make the program more successful?    1. Should an existing in-patient Epic referral be used, or should there be a direct warm-handoff between ED and [HIV CLINIC] staff? 2. What challenges do you anticipate with implementing this workflow? How might they be overcome? 3. What would you like ED providers to know before they begin prescribing PrEP?   Anticipated comfort levels and close-out:   1. If this workflow was implemented as we discussed, how comfortable do you think you would be with this new procedure, from your perspective as [HIV CLINIC] [interviewee’s job title]? Why? 2. Is there anything else you would like me to know? 3. Do you have any colleagues who you think I should talk to about this project? |
| Emergency Department and HIV Clinic Pharmacy Staff | Pre-Exposure Prophylaxis for HIV (PrEP) is a daily pill (or injection administered every two months) that’s 99% effective at preventing HIV. Emergency Departments have been described as promising locations to identify HIV PrEP candidates due to the high volume of patients, many of whom are not engaged in Primary Care and experience risk factors such as injection drug use, transactional sex, and houselessness. We are conducting a needs assessment to evaluate the feasibility of implementing a PrEP initiation and connection-to-care program at the [HOSPITAL] ED. ED providers would identify patients at high risk for HIV, order labs and an initial prescription, and provide a direct referral to ongoing services. The purpose of this interview is to get your perspective on the potential creation and implementation of the program. With your consent, our conversation today will be transcribed and recorded. Thank you for your participation in this study.  Background:   1. Tell me about your role at [HOSPITAL]. 2. How long have you been in practice? 3. How long have you been working at [HOSPITAL]?   PrEP experience: Now, let’s talk about PrEP.   1. Have you ever been involved in the prescribing of same-day initiated or meds medication?    1. Have you ever been involved in the prescribing of same-day initiated PrEP? 2. Have you or a colleague ever received a referral from the ED for PrEP or HIV care? If yes, how did it go? 3. How helpful do you think ED-initiated PrEP would be for at-risk patients who present to the ED?   Creation and implementation of workflow and inter-professional collaboration: Imagine that we develop a standard workflow for prescribing PrEP in the ED.   1. What aspects of a PrEP initiation program would make the program more successful? 2. What challenges do you anticipate with implementing this workflow? How might they be overcome? 3. What would you like ED providers to know before they begin prescribing PrEP?   Anticipated comfort levels and close-out:   1. If this workflow was implemented as we discussed, how comfortable do you think you would be with this new procedure, from your perspective as [HIV CLINIC] [interviewee’s job title]? Why? 2. Is there anything else you would like me to know? 3. Do you have any colleagues who you think I should talk to about this project? |

**Supplemental Table 2:** Study’s verbal consent script

| Verbal Consent Script |
| --- |
| Hello, my name is [INTERVIEWER NAME] from the [INTERVIEWER INSTITUTION]. I’d like to ask you to participate in a research study about creating and implementing an HIV prevention medication workflow in the Emergency Department.  If you agree to be in this study, we will ask you participate in a confidential, one-on-one interview because you are an essential member of the proposed workflow. Interviews will take place on a videoconference at a time of your convenience. We expect that the interview will take approximately thirty minutes.  Being in this study is optional, and you can tell me if you want to stop being in the study at any time. You will receive a $25 gift card for participating. Your decision to participate, withdraw or not answer some questions will have no bearing on your employment or academic standing. You can choose to skip any questions. To protect your confidentiality, your name and other identifying information will not be recorded on any study documents. Numeric identifiers will be used in all notes and interview transcriptions rather than names. Results will appear only in aggregated form, and nothing will be attributed to any identifiable individual. The interview recordings will be stored in a secure folder.  Do you have any questions about the study?  Would you like to participate?  Do you consent to be recorded?  If you have questions about this study in the future, you can contact [PRINCIPAL INVESTIGATOR] at  [PRINCIPAL INVESTIGATOR EMAIL]. If you have questions or concerns about your rights as a research participant, you can call the [INSTITUTION’S INSTUTIONAL REVIEW BOARD] at [INSTUTIONAL REVIEW BOARD PHONE NUMBER]. Our IRB number is [REFERENCE NUMBER]. |
